# Magnetic resonance imaging radiomics for predicting pathologic complete response to neoadjuvant chemotherapy in breast cancer

**DOI:** 10.1590/1806-9282.20260878

**Published:** 2026-11-02

**Authors:** Emin Demirel, Huseyin Coskun

**Affiliations:** 1 Afyonkarahisar University of Health Sciences, Faculty of Medicine, Department of Radiology – Afyonkarahisar, Turkey.

**Keywords:** Breast cancer, Neoadjuvant chemotherapy, Pathologic complete response, Radiomics, Magnetic resonance imaging, Machine learning

## Abstract

**OBJECTIVE:**

The aim of this study was to develop and validate machine learning models integrating clinical and pre-treatment dynamic contrast-enhanced magnetic resonance imaging radiomic features for predicting pathologic complete response to neoadjuvant chemotherapy in breast cancer.

**METHODS:**

This retrospective study analyzed 299 breast cancer patients from the publicly available Duke Breast Cancer magnetic resonance imaging dataset who received neoadjuvant chemotherapy. From pre-treatment dynamic contrast-enhanced magnetic resonance imaging, 534 IBSI-compliant radiomic features were extracted. A five-method consensus feature selection pipeline retained 24 features. Notably, three prediction models were developed: clinical-only (Model A), radiomic-only (Model B), and combined clinical–radiomic (Model C). In total, seven machine learning algorithms were compared using 20 repeated five-fold stratified cross-validation with SMOTE applied within each training fold and assessed on a held-out test set (30%, n=90). Performance was evaluated using AUC with bootstrap confidence intervals, calibration (Brier score), and decision curve analysis.

**RESULTS:**

The pathologic complete response rate was 20.4% (61/299). The combined model (Gradient Boosting) achieved the highest test AUC of 0.723 (95%CI 0.595–0.835), with sensitivity of 0.333 and specificity of 0.833. DeLong tests revealed no statistically significant differences between model pairs (all p>0.05). Decision curve analysis demonstrated the highest net benefit for the combined model across 10–50% threshold probabilities.

**CONCLUSION:**

The combined clinical–radiomic model demonstrated modest discriminative ability for pathologic complete response prediction. The limited sensitivity suggests potential utility as a rule-out test to identify patients unlikely to achieve pathologic complete response. These hypothesis-generating findings require external validation in independent multi-center cohorts.

## INTRODUCTION

Breast cancer is the most frequently diagnosed malignancy and leading cause of cancer-related mortality among women worldwide, with an estimated 2.3 million new cases annually^1^. Neoadjuvant chemotherapy (NAC) has become the standard of care for locally advanced and high-risk early stage breast cancers, particularly within triple-negative (TNBC) and HER2-positive subtypes^2,3^, where pathologic complete response (pCR) rates have been reported to be highest^4^. NAC facilitates tumor downstaging, enabling conversion from mastectomy to breast-conserving surgery, and provides in vivo assessment of chemosensitivity.

pCR, defined as the absence of residual invasive carcinoma (ypT0/Tis ypN0) at surgical resection, serves as a well-established surrogate endpoint for long-term oncologic outcomes^5,6^. Patients achieving pCR demonstrate significantly improved event-free and overall survival, with the strongest association in aggressive subtypes^7^. However, pCR rates remain variable (20–40%), and many patients experience chemotherapy toxicity without therapeutic benefit^8^. This underscores the need for reliable pre-treatment biomarkers capable of identifying likely responders.

Conventional clinicopathologic parameters, including tumor grade, hormone receptor status, and Ki-67 index, have demonstrated limited predictive accuracy for pCR^9^. These markers are constrained by their reliance on small biopsy specimens that fail to represent intratumoral heterogeneity^10^. Imaging-based approaches, particularly MRI, have shown promise in this context. Dynamic contrast-enhanced MRI (DCE-MRI) is especially useful in women with dense breasts, where mammographic sensitivity is reduced^11^.

Radiomics, the high-throughput extraction of quantitative features from medical images, has emerged as a promising approach for non-invasive tumor phenotyping^12,13^. DCE-MRI represents the reference-standard imaging modality in breast oncology, offering superior soft-tissue contrast and rich spatiotemporal data^14^. Quantitative radiomic features from pre-treatment DCE-MRI have demonstrated potential in capturing tumor pathophysiology and microarchitectural heterogeneity^15^.

Despite this promise, methodological challenges have impeded clinical translation, including heterogeneous study designs, inconsistent feature extraction protocols, and overfitted models^16,17^. A comprehensive understanding of how molecular subtypes relate to imaging phenotypes is critical for interpreting radiomic findings^18^. The present study leverages the publicly available Duke Breast Cancer MRI dataset^19^ to develop and validate prediction models integrating clinical and radiomic features for pCR prediction, in accordance with TRIPOD guidelines^20^ and IBSI feature standardization^21^.

## METHODS

### Study population and design

This retrospective study utilized the Duke Breast Cancer MRI dataset from The Cancer Imaging Archive^19^. From 922 female patients, 299 who received NAC with available pathologic response data were included. The primary outcome was pCR, defined as ypT0/Tis ypN0 (pCR: n=61, 20.4%; non-pCR: n=238, 79.6%). The dataset is publicly available and de-identified. Written informed consent was obtained from all participants at the time of original data collection. This research complied with the Declaration of Helsinki.

### Magnetic resonance imaging acquisition and radiomic feature extraction

All examinations were performed using 1.5T or 3.0T MRI scanners with dedicated breast coils. DCE-MRI included pre-contrast T1-weighted and dynamic post-contrast acquisitions after gadolinium-based contrast agent administration (0.1 mmol/kg, 2 mL/s). Tumor regions of interest were delineated by experienced breast radiologists. A comprehensive set of 534 radiomic features was extracted using a custom Python pipeline built upon PyRadiomics (version 3.0.1, IBSI-compliant), encompassing tumor morphology, first-order statistics, gray-level co-occurrence matrix texture features, kinetic map features, and spatial autocorrelation metrics. Image preprocessing included z-score normalization with fixed bin width discretization.

### Feature selection

Features underwent systematic quality control: removal of constant, near-zero variance, and highly correlated features (Pearson |r|>0.95), reducing the set from 534 to 286. A five-method consensus approach was implemented: (1) Mann-Whitney U test with FDR correction (p<0.05), (2) LASSO logistic regression (lambda.1se), (3) mutual information ranking (top 50), (4) recursive feature elimination with five-fold cross-validation, and (5) random forest importance (top 50). Features selected by at least two of the five methods were retained (n=24). When features were selected by exactly two methods (representing 11 of the 24 retained features), the direction of association with pCR was confirmed to be consistent between the two methods. This consensus threshold was set a priori to balance dimensionality reduction with retention of potentially informative features.

### Model development

Notably, three prediction models were developed: Model A (clinical-only, 12 variables: age, ER status, PR status, HER2 status, tumor grade, HR subtype, TNBC status, tumor size, clinical stage, Ki-67 index, lymph node status, menopausal status), Model B (radiomic-only, 24 features), and Model C (combined, 36 features). In total, seven machine learning algorithms were evaluated: L2-regularized logistic regression (C=1.0), elastic net (alpha=0.5, l1_ratio=0.5), random forest (n_estimators=500), XGBoost (n_estimators=100, max_depth=4), LightGBM (n_estimators=100, num_leaves=31), SVM-RBF (C=1.0, gamma=”scale”), and gradient boosting (n_estimators=100, max_depth=3). The dataset was partitioned into training (70%, n=209) and test (30%, n=90) sets with stratified sampling. SMOTE (k-neighbors=5)^20^ was applied within each cross-validation training fold to prevent data leakage. Hyperparameter tuning used grid search with five-fold cross-validation.

### Statistical analysis

Model performance was evaluated using AUC with 95%CIs from 1,000 bootstrap resamples, sensitivity, specificity, PPV, NPV, calibration, Brier score (ranging from 0 to 1, where lower values indicate better calibration), and decision curve analysis^21^. The Brier score represents the mean squared difference between predicted probabilities and observed outcomes; a score of 0 indicates perfect prediction, while 0.25 reflects a non-informative model for binary outcomes with approximately 50% prevalence. Model comparison used the DeLong test^22^. Internal validation employed 20 repeated five-fold stratified cross-validations. All analyses used Python 3.10 with scikit-learn (1.1.0), XGBoost (1.6.0), LightGBM (3.3.0), and SHAP (0.41.0). Statistical significance was set at two-sided p<0.05. The code underlying this analysis is available from the corresponding author upon reasonable request.

## RESULTS

### Study population and clinical characteristics

Among 299 patients, 61 (20.4%) achieved pCR. ER-negative status (p=0.001), PR-negative status (p<0.001), HER2-positive status (p=0.006), and triple-negative subtype (p<0.001) were significantly associated with higher pCR rates (Table 1). The molecular subtype distribution was: HR+/HER2- (46.5%), HR+/HER2+ (17.7%), HR-/HER2+ (8.7%), and TNBC (27.1%). TNBC accounted for 36.1% of pCR cases despite comprising only 27.1% of the cohort, consistent with the known association between TNBC and higher chemosensitivity.

**Table 1:** Patient demographics and clinical characteristics.

Variable	All (n=299)	pCR (n=61)	Non-pCR (n=238)	p-value
Age, years, median (IQR)	48 (42–57)	47 (38–55)	49 (42–58)	0.062
ER positive, n (%)	184 (61.5)	26 (42.6)	158 (66.4)	0.001
PR positive, n (%)	151 (50.5)	14 (23.0)	137 (57.6)	<0.001
HER2 positive, n (%)	79 (26.4)	25 (41.0)	54 (22.7)	0.006
Grade 1/2/3	6/30/263	0/3/58	6/27/205	0.137
HR+/HER2-, n (%)	139 (46.5)	14 (23.0)	125 (52.5)	
HR+/HER2+, n (%)	53 (17.7)	15 (24.6)	38 (16.0)	
HR-/HER2+, n (%)	26 (8.7)	10 (16.4)	16 (6.7)	
TNBC, n (%)	81 (27.1)	22 (36.1)	59 (24.8)	<0.001

p-values from Mann-Whitney U test (continuous) and chi-square test (categorical). ER: estrogen receptor; PR: progesterone receptor; HER2: human epidermal growth factor receptor 2; HR: hormone receptor; TNBC: triple-negative breast cancer; IQR: interquartile range; pCR: pathologic complete response.

### Feature selection

The consensus feature selection identified 24 features selected by at least two methods. In total, three features were selected by all five methods: Tumor Grade (Mitotic), Change in Variance of Uptake (Tumor), and SER Map Max Probability (Tumor). Of the remaining features, 11 were selected by exactly two methods (with consistent direction of association confirmed), 7 by three methods, and 3 by four methods. The radiomic features span first-order statistics (n=8), texture/GLCM features (n=7), kinetic features (n=6), and morphological features (n=3).

### Predictive model performance

The combined model (Model C, Gradient Boosting) achieved the highest test AUC of 0.723 (95%CI 0.595–0.835), with sensitivity of 0.333 and specificity of 0.833 (Table 2). The clinical-only model (Model A) achieved AUC of 0.700 (95%CI 0.581–0.819), while the radiomic-only model (Model B) achieved AUC of 0.615 (95%CI 0.468–0.748). Pairwise DeLong tests revealed no statistically significant differences between any model pair (all p>0.05). Internal validation via 20 repeated five-fold cross-validation yielded mean AUCs of 0.694 (SD: 0.091), 0.749 (SD: 0.080), and 0.764 (SD: 0.076) for Models A, B, and C, respectively.

**Table 2:** Test set performance of best-performing models.

Model	Algorithm	AUC (95%CI)	Sensitivity	Specificity	PPV	Brier
Clinical (A)	Logistic Reg.	0.700 (0.581–0.819)	0.667	0.681	0.343	0.209
Radiomic (B)	Gradient Boost.	0.615 (0.468–0.748)	0.222	0.806	0.222	0.214
Combined (C)	Gradient Boost.	0.723 (0.595–0.835)	0.333	0.833	0.333	0.190

AUC: area under the receiver operating characteristic curve; CI: confidence interval; PPV: positive predictive value; Brier: Brier score. All metrics were calculated at the Youden J-statistic optimal threshold. Models: A=clinical-only (12 variables), B=radiomic-only (24 features), C=combined (36 features).

The combined model achieved a Brier score of 0.190 (Model A: 0.209, Model B: 0.214), indicating acceptable calibration. Decision curve analysis demonstrated that the combined model provided the highest net benefit across threshold probabilities of 10–50%. Figure 1 presents the ROC curves for all three models.

**Figure 1:**
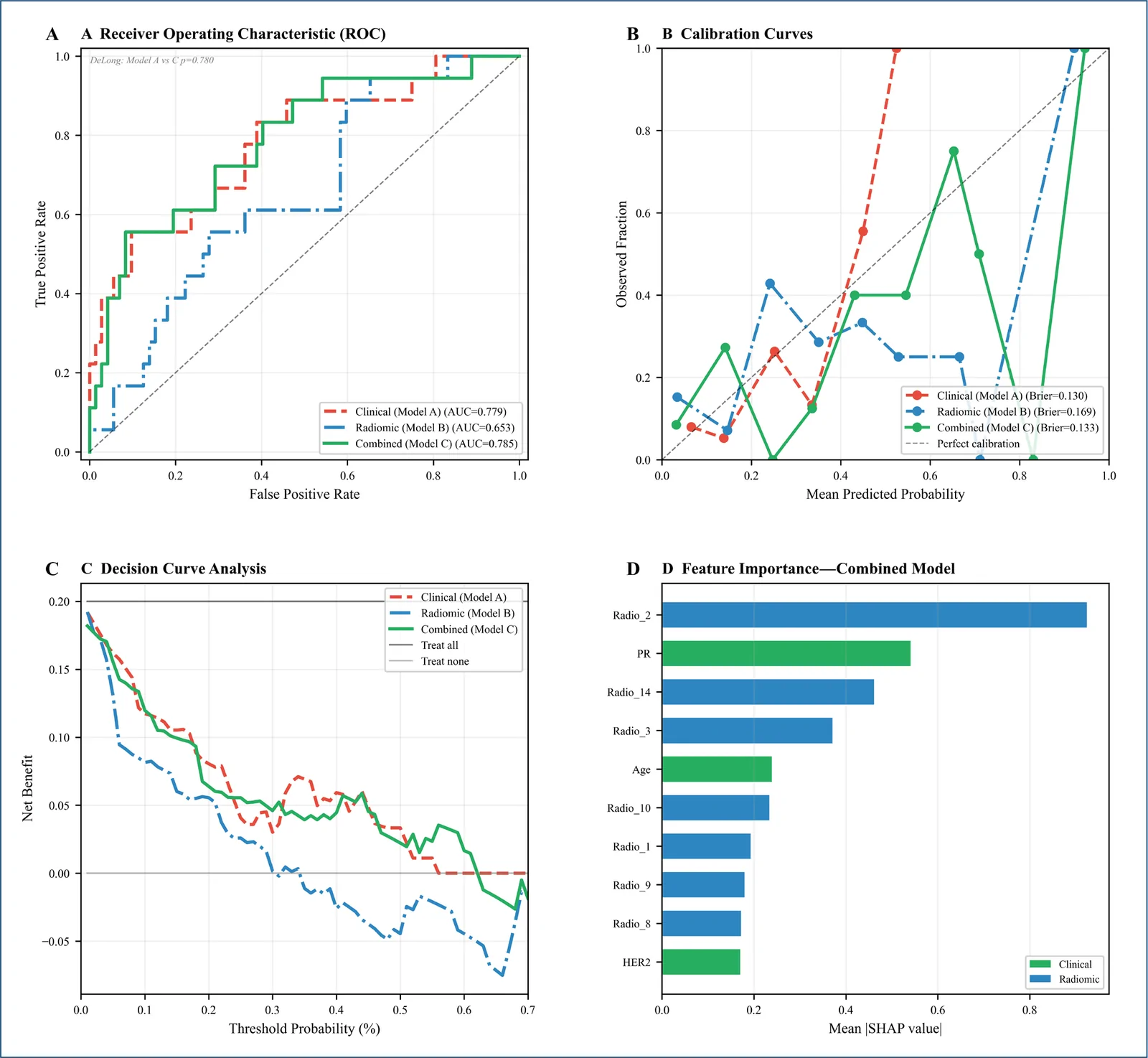
Four-panel multipanel figure. (A) Receiver operating characteristic (ROC) curves for the three prediction models on the held-out test set. The combined model (Model C, Gradient Boosting) achieved the highest AUC of 0.723 (95% CI 0.595–0.835). DeLong test results: Model A vs. B: p=0.321; Model A vs. C: p=0.780; Model B vs. C: p=0.153. (B) Calibration curves demonstrating agreement between predicted probabilities and observed outcomes. Brier scores: Model A=0.209, Model B=0.214, Model C=0.190. (C) Decision curve analysis showing net benefit across threshold probabilities. The combined model provided the highest net benefit across 10–50% threshold probabilities. (D) SHAP feature importance (top 10) for the combined model. Radiomic features in blue; clinical features in green.

## DISCUSSION

In this exploratory prediction model study, the combined clinical–radiomic model achieved the numerically highest AUC of 0.723 for pCR prediction; however, the incremental improvement over the clinical-only model was not statistically significant (DeLong p=0.780). This null finding is critical and must be interpreted with caution. The wide confidence interval reflects limited statistical power (n=90 test set, event rate ~20%), precluding definitive conclusions. Post-hoc consideration of sample size suggests that a formal power calculation would require substantially larger cohorts to detect moderate AUC improvements—our study was likely underpowered for this purpose. Prior studies reporting higher AUCs (0.65–0.92)^15,23^ may reflect methodological differences, including smaller sample sizes, single-method feature selection, and less rigorous validation approaches. Our findings should be considered hypothesis-generating rather than confirmatory.

The biological interpretability of selected radiomic features provides important insights. Signal enhancement ratio (SER) and peak enhancement (PE) are directly related to tumor angiogenesis and microvascular perfusion dynamics. High SER map max probability within the tumor reflects rapid contrast uptake and washout, indicative of abnormal vascular architecture driven by VEGF-mediated neovascularization. This high-perfusion profile may facilitate chemotherapeutic agent penetration, increasing pCR likelihood. Similarly, increased variance in tumor uptake reflects spatial heterogeneity, potentially harboring subclones with varying chemosensitivity.

A critical methodological consideration is potential confounding by molecular subtype. Our analysis shows that TNBC and HER2+ subtypes are strongly associated with pCR (Table 1), and these subtypes also have distinct imaging characteristics on DCE-MRI. It is possible that radiomic features partly capture subtype-specific imaging phenotypes rather than providing entirely independent predictive information. This is consistent with our observation that the clinical-only model (which includes subtype variables) performed comparably to the combined model. Future studies employing larger, subtype-stratified samples are needed to determine whether radiomic features provide independent predictive value beyond clinical subtype classification.

The clinical translation of our model must be carefully considered in light of its operating characteristics. With a sensitivity of 0.333 at the Youden-optimal threshold, the model would miss two-thirds of true pCR cases if used as a standalone diagnostic test. However, the high specificity (0.833) and reasonable net benefit on DCA suggest potential clinical utility as a rule-out test—identifying patients with very low probability of pCR who might benefit from alternative therapeutic strategies or closer monitoring during NAC. At the current performance level, we envision this model as a decision-support tool rather than a replacement for clinical judgment. A higher sensitivity threshold (e.g., targeting 0.80 sensitivity) could be clinically justified for specific scenarios but would substantially reduce specificity.

From a methodological perspective, our study distinguishes itself through strict adherence to TRIPOD guidelines and IBSI standards. The radiomic-only model exhibited a substantial gap between cross-validation AUC (0.749) and test AUC (0.615), with a drop of 0.134 AUC points, demonstrating persistent challenges of overfitting in high-dimensional radiomic studies despite multi-method feature selection. This gap, which reflects the broader challenge of radiomic feature generalizability, suggests that handcrafted radiomic features may capture dataset-specific noise rather than truly generalizable biological signals. Our five-method consensus approach reduced dimensionality from 534 to 24 features, representing a more robust alternative to single-method selection. Feature selection stability remains a concern: only three features were selected by all five methods, indicating that consensus-based approaches may benefit from bootstrap stability analysis to identify consistently selected features.

Our AUC of 0.723 falls within the moderate range of published studies (0.65–0.92)^15,16,23^. We chose conservative validation with honest performance reporting rather than optimistically biased results. The substantial gap between radiomic-only cross-validation and test performance (0.749 vs. 0.615) highlights a critical challenge in radiomics research: handcrafted features may generalize poorly compared to deep learning approaches that learn task-specific representations.

Several limitations warrant discussion. First, the absence of external validation is the most significant limitation—the Duke dataset is single-center, and no publicly available independent dataset with matched variables and NAC response data was identified for external testing. Second, exclusive use of pre-treatment imaging precludes capturing dynamic changes during NAC (delta-radiomics). Third, low sensitivity (0.333) limits standalone clinical utility. Fourth, inter-reader reproducibility was not independently assessed. Fifth, the non-significant DeLong tests indicate that the incremental value of radiomics over clinical variables was not statistically demonstrated. Sixth, our reliance on handcrafted radiomic features may not capture the full complexity of tumor biology that deep learning approaches might extract.

Recent work by Cristófalo et al.^4^ provided contemporary data on pCR rates in TNBC, confirming that this subtype achieved the highest response rates and represented the subgroup where radiomic prediction models might be most clinically impactful. Mota et al.^11^ highlighted the importance of advanced imaging in dense breasts, supporting our rationale for MRI-based radiomics. Rivas et al.^24^ demonstrated the relevance of molecular subtype characterization in breast cancer detection, contextualizing our finding that subtype confounds radiomic-signal prediction. Future research should include external multi-center validation, delta-radiomics analysis, deep learning approaches, and multi-omics integration^24,25^.

## CONCLUSION

The combined clinical–radiomic model achieved the numerically highest predictive performance for pCR (test AUC=0.723, 95%CI 0.595–0.835). However, the incremental value of radiomic features over clinical variables alone was not statistically significant (DeLong p>0.05), and low sensitivity (0.333) limits standalone clinical deployment. Given its high specificity, the model may have utility as a rule-out test to identify patients unlikely to benefit from standard NAC who might be candidates for alternative strategies or clinical trials. These findings are hypothesis-generating and require external validation in independent, multi-center cohorts before any clinical implementation.

## Data Availability

The datasets generated and/or analyzed during the current study are available from the corresponding author upon reasonable request.

## References

[1] Sung H, Ferlay J, Siegel RL, Laversanne M, Soerjomataram I, Jemal A (2021). Global cancer statistics 2020: GLOBOCAN estimates of incidence and mortality worldwide for 36 cancers in 185 countries. CA Cancer J Clin.

[2] Cortazar P, Zhang L, Untch M, Mehta K, Costantino JP, Wolmark N (2014). Pathological complete response and long-term clinical benefit in breast cancer: the CTNeoBC pooled analysis. Lancet.

[3] Kaufmann M, Minckwitz G, Mamounas EP, Cameron D, Carey LA, Cristofanilli M (2012). Recommendations from an international consensus conference on the current status and future of neoadjuvant systemic therapy in primary breast cancer. Ann Surg Oncol.

[4] Cristófalo MM, Maesaka JY, Pereira DA, Nóbrega GB, Reis YN, Soares JM (2026). Neoadjuvant chemotherapy in triple negative breast cancer: a systematic review of breast and node pathologic response. J Surg Oncol.

[5] Spring LM, Fell G, Arfe A, Sharma C, Greenup R, Reynolds KL (2020). Pathologic complete response after neoadjuvant chemotherapy and impact on breast cancer recurrence and survival: a comprehensive meta-analysis. Clin Cancer Res.

[6] Minckwitz G, Untch M, Blohmer JU, Costa SD, Eidtmann H, Fasching PA (2012). Definition and impact of pathologic complete response on prognosis after neoadjuvant chemotherapy in various intrinsic breast cancer subtypes. J Clin Oncol.

[7] Houssami N, Macaskill P, Minckwitz G, Marinovich ML, Mamounas E (2012). Meta-analysis of the association of breast cancer subtype and pathologic complete response to neoadjuvant chemotherapy. Eur J Cancer.

[8] Haque W, Verma V, Butler EB, Teh BS (2018). Response rates and pathologic complete response by breast cancer molecular subtype following neoadjuvant chemotherapy. Breast Cancer Res Treat.

[9] Pilewskie M, Morrow M, Bao T, Eaton A, Patil S, King TA (2016). Predictors of pathologic complete response after neoadjuvant chemotherapy in breast cancer. Ann Surg Oncol.

[10] Marusyk A, Almendro V, Polyak K (2012). Intra-tumour heterogeneity: a looking glass for cancer?. Nat Rev Cancer.

[11] Mota BS, Shimizu C, Reis YN, Gonçalves R, Soares JM, Baracat EC (2025). Dense breasts and women’s health: which screenings are essential?. Clinics (Sao Paulo).

[12] Lambin P, Leijenaar RTH, Deistl TM, Peerlings J, Jong EEC, Timmeren J (2017). Radiomics: the bridge between medical imaging and personalized medicine. Nat Rev Clin Oncol.

[13] Gillies RJ, Kinahan PE, Hricak H (2016). Radiomics: images are more than pictures, they are data. Radiology.

[14] Mann RM, Cho N, Moy L (2019). Breast MRI: state of the art. Radiology.

[15] Braman NM, Etesami M, Prasanna P, Dubchuk C, Gilmore H, Tiwari P (2017). Intratumoral and peritumoral radiomics for the pretreatment prediction of pathological complete response to neoadjuvant chemotherapy based on breast DCE-MRI. Breast Cancer Res.

[16] Liu Z, Li Z, Qu J, Zhang R, Zhou X, Li L (2019). Radiomics of multiparametric MRI for pretreatment prediction of pathologic complete response to neoadjuvant chemotherapy in breast cancer: a multicenter study. Clin Cancer Res.

[17] Saha A, Harowicz MR, Grimm LJ, Kim CE, Ghate SV, Walsh R (2018). A machine learning approach to radiogenomics of breast cancer: a study of 922 subjects and 529 DCE-MRI features. Br J Cancer.

[18] Collins GS, Reitsma JB, Altman DG, Moons KGM (2015). Transparent reporting of a multivariable prediction model for individual prognosis or diagnosis (TRIPOD): the TRIPOD statement. BMJ.

[19] Zwanenburg A, Vallières M, Abdalah MA, Aerts HJWL, Andrearczyk V, Apte A (2020). The image biomarker standardization initiative: standardized quantitative radiomics for high-throughput image-based phenotyping. Radiology.

[20] Chawla NV, Bowyer KW, Hall LO, Kegelmeyer WP (2002). SMOTE: synthetic minority over-sampling technique. J Artif Intell Res.

[21] Vickers AJ, Elkin EB (2006). Decision curve analysis: a novel method for evaluating prediction models. Med Decis Making.

[22] DeLong ER, DeLong DM, Clarke-Pearson DL (1988). Comparing the areas under two or more correlated receiver operating characteristic curves: a nonparametric approach. Biometrics.

[23] Granzier RWY, Nijnatten TJA, Woodruff HC, Smidt ML, Lobbes MBI (2019). Exploring breast cancer response prediction to neoadjuvant systemic therapy using MRI-based radiomics: a systematic review. Eur J Radiol.

[24] Rivas FWS, Gonçalves R, Mota BS, Sorpreso ICE, Toporcov TN, Filassi JR (2024). Comprehensive diagnosis of advanced-stage breast cancer: exploring detection methods, molecular subtypes, and demographic influences — A cross-sectional study. Clinics (Sao Paulo).

[25] Calster B, McLernon DJ, Smeden M, Wynants L, Steyerberg EW (2019). Topic Group “Evaluating Diagnostic Tests and Prediction Models” of the STRATOS Initiative. Calibration: the Achilles heel of predictive analytics. BMC Med.

